# Some Forebodings of Incipient Insanity

**Published:** 1887-05

**Authors:** 


### SOME FOREBODINGS OF INCIPIENT INSANITY.

1. Irritability and tendency to take offense.
2. Moroseness and silence, or sometimes fault-finding with servants.
3. Suspicion and jealousy of best friends.
4. Impairment of memory, forgetting hours of meals.
5. Inattention to exercise and state of bowels.
6. Neglect of personal appearance.
7. Altered facial expression, notably in melancholia, with marked furrows.
8. Prominence and brilliancy of corneæ, in hysterical and puerperal mania.

BODILY SYMPTOMS.

1. Harsh, dry skin, as a rule, though sometimes perspiring.
2. Sometimes a peculiar odor.
3. Coated tongue, with offensive breath.
4. Constipation and feeble circulation.
5. Headache and pallor of face.
6. Sexual appetite, either in abeyance or abnormally strong.
7. Frequent suppression of menses in females.
8. Subjected deafness, or abnormal auditory sensations.
9. Altered conversational style, and talking to ones-self.
10. Delusions and illusions later on.

[Abridged from a paper by Dr. Sutherland, lecturer on insanity, Westminster Hospital, London.]

